# Chondrocyte-Specific Knockout of TSC-1 Leads to Congenital Spinal Deformity in Mice

**DOI:** 10.1155/2017/8215805

**Published:** 2017-04-24

**Authors:** Cheng Yang, Yuhui Chen, Zhen Li, He Cao, Keming Chen, Pinglin Lai, Bo Yan, Bin Huang, Jiajun Tang, Shicai Fan, Daozhang Cai, Dadi Jin, Xiaochun Bai, Rongping Zhou

**Affiliations:** ^1^Academy of Orthopedics of Guangdong Province, Department of Orthopedic Surgery, The Third Affiliated Hospital of Southern Medical University, Guangzhou 510630, China; ^2^Department of Anesthesia, Zhongnan Hospital of Wuhan University, Wuhan 430071, China; ^3^Department of Pathology, Sun Yat-sen University Cancer Center, Guangzhou 510060, China; ^4^Department of Cell Biology, School of Basic Medical Science, Southern Medical University, Guangzhou 510515, China; ^5^Department of Orthopedics, The Second Affiliated Hospital of Nanchang University, Nanchang, Jiangxi 330006, China

## Abstract

Congenital spinal deformity is the most severe clinical orthopedic issue worldwide. Among all the pathological processes of congenital spinal deformity, the imbalance of endochondral ossification is considered to be the most important developmental cause of spinal dysplasia. We established chondrocyte-specific TSC-1 knockout (KO) mice to overactivate the energy metabolic component, mammalian target of rapamycin complex 1 (mTORC1), and measured the spinal development by general, imaging, histological, and Western-blot assessments. In addition to skeletal dysplasia, the KO mice displayed severe congenital spinal deformity and significant intervertebral disc changes. This study suggests that, in the process of endochondral ossification, excessive activation of mTORC1 signaling in chondrocytes induces obvious spinal deformity, and the chondrocytes may be the cell type responsible for congenital spinal deformity.

## 1. Introduction

Congenital spinal deformity is the most severe clinical orthopedic issue worldwide. For spontaneous chest dysplasia and functional loss of respiratory organs, congenital spinal deformity is considered to be a fatal disorder with >60% mortality [1]. Nevertheless, due to the molecular mechanism involved, the pathological process of congenital spinal deformity is not fully understood. Currently, the only sufficient therapy for congenital spinal deformity is orthopedic surgery. Therefore, the relevant genomic mutations need to be determined for congenital spinal deformity.

Endochondral ossification, a process in which bone formation initiates from a cartilage intermediate, is crucial for skeletal development [2, 3]. In this process, periodic activation of multiple signaling pathways plays a significant role and disturbance of these pathways leads to skeletal disorders like scoliosis and kyphosis [4–9]. Nevertheless, the molecular mechanisms responsible for spinal dysplasia are largely unknown. Recent studies have shown that mammalian target of rapamycin (mTOR) plays a vital role in cartilage growth and skeletal development. mTOR and mTOR complex 1 (mTORC1) knockout (KO) mice show delayed embryonic bone growth and cartilage hypertrophy, which finally blocks bone formation [10]. In addition, it has been shown that mTOR activation is necessary for chondrogenesis and cartilage growth, as well as skeletal development [11]. Rokutanda et al. [12] have found that, in bone development, the AKT–mTOR signaling pathway plays a regulatory role in chondrogenesis and cartilage hypertrophy [13]. Rapamycin (mTOR inhibitor) retards bone formation through blocking angiogenesis in the growth plate of mammals [14].

In the present study, we aimed to determine the potential role of mTOR in spinal development. The chondrocyte-specific TSC1 (upstream inhibitor of mTORC1) KO mice were used to measure the effects of spinal formation followed by overactivation of mTORC1.

## 2. Results

### 2.1. Postnatal Observation of Wild-Type and TSC-1 KO Mice

**Figure 1:**
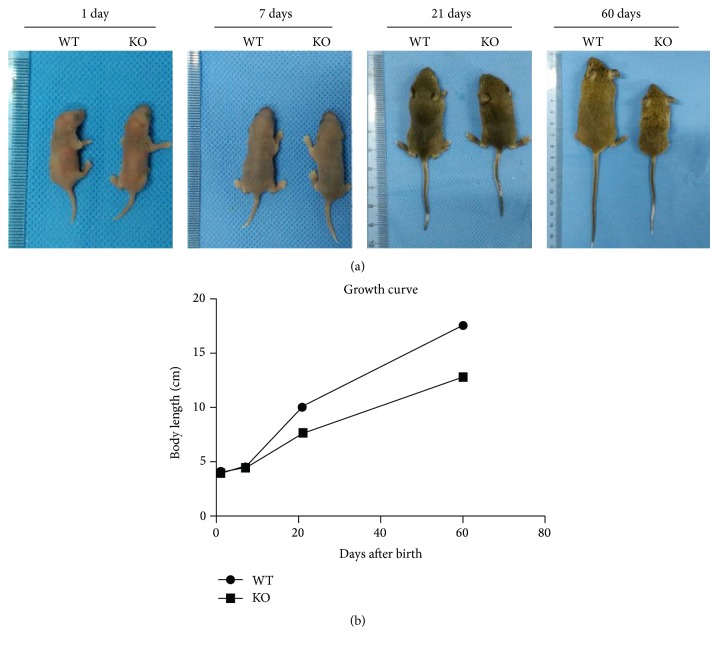
(a) General observations of TSC-1 null and WT mice at 1, 7, 28, and 60 days postnatally. (b) Statistical analysis of body length of TSC-1 null and WT mice at 1, 7, 28, and 60 days postnatally.

The body length of wild-type (WT) (*n* = 10) and KO (*n* = 10) mice was measured at 1, 7, 21, and 60 days postnatally. Although no general alterations were observed between WT and KO mice at 1 and 7 days (Figures 1(a) and 1(b); *p* > 0.05), a significant reduction in body length and weight was seen in KO mice at 21 and 60 days when compared to WT mice (Figures 1(a) and 1(b); *p* < 0.001).

### 2.2. TSC-1 Null Mice Displayed Congenital Spinal Deformity

**Figure 2:**
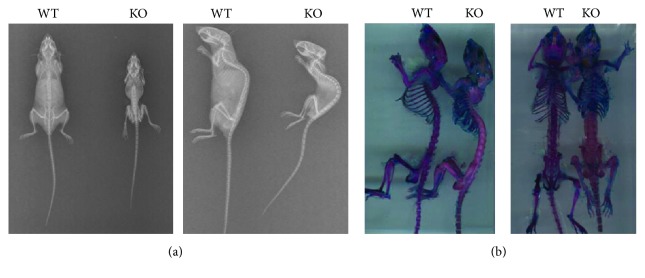
(a) X-ray images of TSC-1 null and WT mice at 60 days postnatally. (b) Whole skeletal stains of TSC-1 null and WT mice at 60 days postnatally.

**Figure 3:**
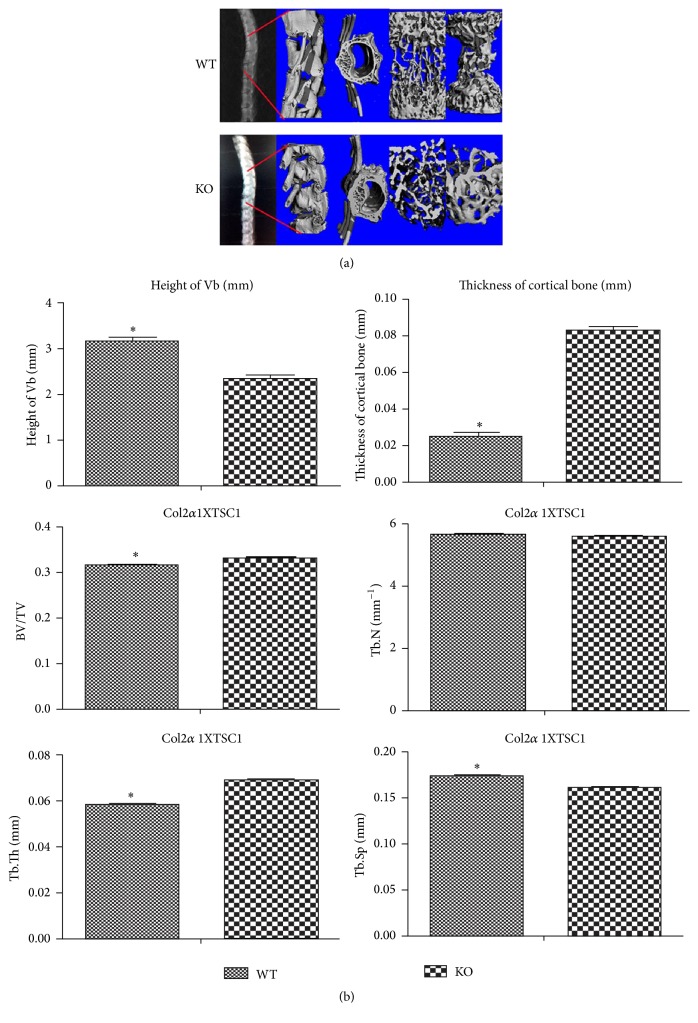
(a) Micro-CT analysis of TSC-1 null and WT mice at 60 days postnatally. (b) Statistical analysis of micro-CT parameters of TSC-1 null and WT mice at 1, 7, 28, and 60 days postnatally. *∗* represents the statistical difference between two groups.

To compare the spinal development of WT (*n* = 10) and KO (*n* = 10) mice, X-ray and micro-computed tomography (CT) were used to measure the spine of each mouse at 60 days postnatally. The KO mice exhibited smaller and shorter vertebrae when compared to WT mice, although no disc alteration was observed (Figures 2(a) and 3(a)). However, micro-CT analysis suggested enhancement of the thickness of cortical bone and density of trabecular bone in KO mice compared with WT mice. KO mice showed loss of intervertebral space and congenital spinal canal stenosis (Figure 3(b)).

To measure cartilage formation of KO mice, whole skeleton staining was performed at 60 days postnatally. In KO mice, significant enlargement of costal cartilage and immature bony structure of the ribs was observed. Moreover, classic flat chest and thoracolumbar kyphosis were present in all KO mice, while no deformity was observed in WT mice at 60 days after birth (Figure 2(b)). These data suggest that overactivation of mTORC1 in chondrocytes may be relevant to congenital spinal deformities in mammals.

### 2.3. TSC-1 Null Mice Showed Intervertebral Disc Dysplasia

**Figure 4:**
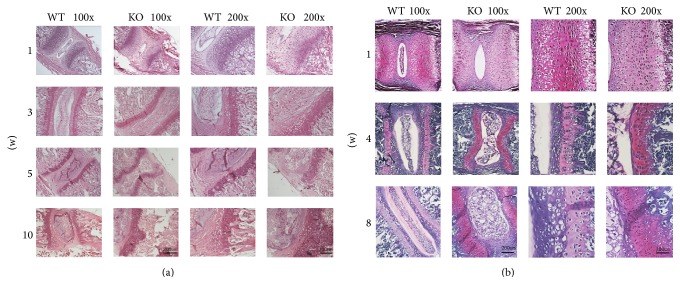
(a) Vertebral staining (HE) of TSC-1 null and WT mice at 1, 3, 5, and 10 weeks postnatally. (b) Vertebral staining (safranin O-toluidine blue) of TSC-1 null and WT mice at 1, 4, and 8 weeks postnatally.

Hematoxylin and eosin (HE) staining of lumbar vertebrae was used to measure the disc development of KO and WT mice at 1, 3, 5, and 10 weeks postnatally and safranin O-toluidine blue staining of lumbar vertebrae was used at 1, 4, and 8 weeks postnatally as well. The disc height of KO mice was significantly reduced after 4 and 8 weeks when compared to WT mice (Figures 4(a) and 4(b)). The classic structure of intervertebral discs, nucleus pulposus, and annulus fibrosus was absent in the intervertebral discs of KO mice at 4 and 8 weeks after birth (Figure 4(b)). Although no change in disc height was observed between the 2 groups at 1 week postnatally, the primary ossification center of KO mice was abnormal (Figures 4(a) and 4(b)). These results suggest that, in addition to the vertebral deformity, the intervertebral discs of KO mice showed signs of dysplasia as well.

Compared to WT mice, high magnification showed marked elevation of chondrogenesis in the growth plate of KO mice, but rarely hypertrophic chondrocytes (Figures 4(a) and 4(b)). It is well known that hypertrophy and nonapoptotic physiological cell death of chondrocytes are vital processes of endochondral ossification [15, 16]; therefore, we suppose that this pathological alteration is a major response in the spinal deformity of KO mice.

### 2.4. Rapamycin Rescued Congenital Spinal Deformity in KO Mice

**Figure 5:**
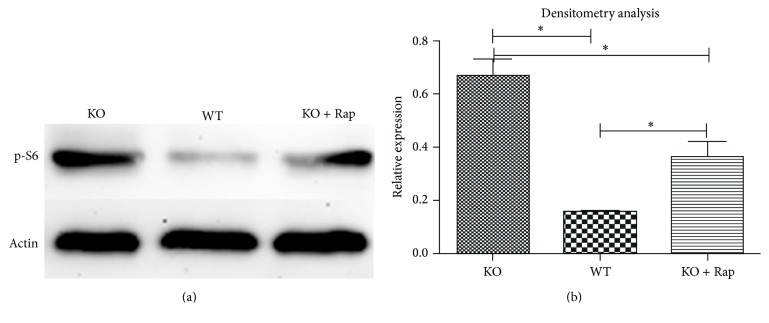
(a) Western-blot results of Ps6 expression in cartilage of WT, KO, and KO + rapamycin mice. (b) Statistical analysis of Ps6 expression in cartilage of WT, KO, and KO + rapamycin mice. *∗* represents the statistical difference between two groups.

KO mice were gavaged with rapamycin (mTORC1-specific inhibitor) by daily intragastric administration after ablactation (*n* = 8). To measure the mTORC1 signaling of KO mice after rapamycin intake, cartilage phosphorylated s6 (the downstream marker of mTORC1 signaling) expression was revealed in WT, KO, and KO + rapamycin mice, respectively. Results showed that the highest level of Ps6 expression was performed in KO mice as the lowest level of Ps6 was present in WT mice; meanwhile, when compared to KO mice, the reduction of Ps6 expression was significant (Figures 5(a) and 5(b)). These data suggested that rapamycin intake can inhibit the overactivated mTORC1 signaling in the cartilage of KO mice.

**Figure 6:**
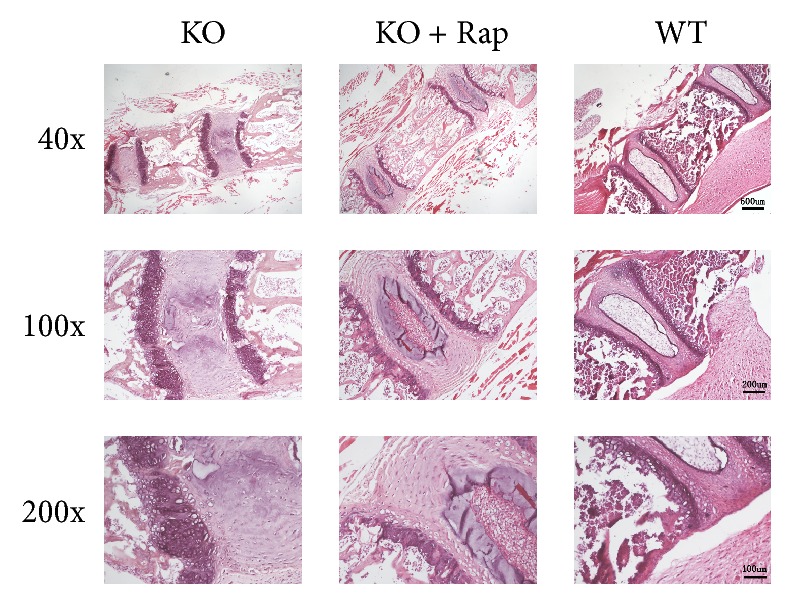
Vertebral staining (HE) of TSC-1 null mice, TSC-1 null mice treated with rapamycin, and WT mice.

After 4 weeks of rapamycin administration, no deformity was seen in KO mice. HE staining of lumbar vertebrae showed that the thickness of cortical bone and density of trabecular bone decreased in KO mice, as well as reduction of disc height (Figure 6). As the classic structure of intervertebral discs, nucleus pulposus, and annulus fibrosus was absent in the intervertebral discs of KO mice at 4 weeks after birth, the formation of nucleus pulposus and annulus fibrosus was restored in KO mice after rapamycin application (Figure 6). Hypertrophic chondrocytes and primary ossification center were observed in each intervertebral disc of KO mice, although there was still a delay in spinal development (Figure 6). These results suggest that the congenital spinal deformity of chondrocyte-specific TSC-1 KO mice was derived from overactivation of mTORC1 signaling.

## 3. Discussion

Recently, numerous studies have measured the potential response genetic mutant of spinal deformity [1, 14, 17]. Deleted growth factor related factor like fibroblast growth factor receptors selectively leads to scoliosis in mice, and the mutant genes related to growth factors are observed in patients with spinal deformity [17, 18]. As a pivotal metabolic component of mammals, mTORC1 signaling is tightly correlated with skeletal development and bone metabolism [10]. Nevertheless, to the best of our knowledge, no investigation has measured the potential role of mTOR signaling in spinal deformity.

In congenital spinal deformity, for both scoliosis and kyphosis, the cell types that contribute to the developmental deformity of vertebrae are still unclear, although several tissue types are involved in formation of the spinal structure, including bone, cartilage, and blood vessels [10]. In the endochondral ossification of the spine, the proliferation and hypertrophy of chondrocytes play a key role and are responsible for vertebral and disc formation. Therefore, in the present study, we overactivated mTORC1 in chondrocytes of mice and measured the spinal development of TSC-1 KO mice. Overactivation of mTORC1 resulted in congenital spinal deformity in mice and mTORC1 inhibitor (rapamycin) rescued this deformity.

Our recent study showed that although overactivation of mTORC1 in preosteoblasts through TSC-1 KO resulted in osteosclerosis and skeletal dysplasia in mice, no significant spinal deformity was present [19]. Nevertheless, in the present study, in addition to congenital skeletal dysplasia, overactivation of mTORC1 in chondrocytes led to spinal kyphosis and abnormal intervertebral discs. This phenomenon suggests that the target cell types that moderate spinal deformity are chondrocytes rather than bone cells (pre- and mature osteoblasts). Therefore, the major views of spinal deformity should change into the chondrocytes and the progress of cartilage formation rather than bony formation in clinical area, especially.

In this investigation, the mTORC1 inhibitor rapamycin partly rescued the skeletal dysplasia and corrected the spinal deformity of TSC-1-specific KO mice. However, an interesting observation in our previous study is that downregulation of mTORC1 in chondrocytes by KO of mTORC1 component (Raptor) also resulted in skeletal dysplasia, although no marked spinal deformity occurred [17]. These results suggest that mTORC1 signaling is the major moderator in complex signaling crosstalk and that inhibition and overactivation can induce skeletal malformation. Similar to our contradictory results, knockdown of mTORC1 in osteoblasts or bone marrow stromal cells leads to inhibition or acceleration of osteogenesis [11, 13]. Although these inconsistencies have not been resolved, the regulatory role of mTORC1 in skeletal development was identified, and therefore, the mechanism of the skeletal modulation by mTORC1 needs further research.

To the best of our knowledge, this is the first report of the role of energy metabolism in the pathological process of spinal deformity. In the process of endochondral ossification, excessive activation of mTOR signaling in chondrocytes induces obvious systemic dysplasia and spinal deformity. Our study suggests a novel potential pathological cause of spinal deformity and identifies the involved cell type as the chondrocytes. Although the mechanism needs to be clarified, our results provide a new insight into congenital spinal deformity.

## 4. Materials and Methods

### 4.1. Generation of Chondrocyte-Specific TSC1 KO

To delete TSC1 specifically in chondrocytes, TSC1flox/−; Col2*α*1-Cre were generated by crossing TSC1 floxed mice (TSC1flox/flox, Jackson Lab, #005680), TSC1 heterozygous mice (TSC1+/−), and Col2*α*1-Cre (Jackson Lab, #006774) transgenic mice. TSC1+/−; Col2*α*1-Cre male mice were crossed with TSC1flox/flox female mice. We used TSC1flox/flox; Col2*α*1-Cre as experimental mice (KO) and TSC1+/+; Col2*α*1-Cre as littermate control mice (WT). The newborn mice were analyzed by polymerase chain reaction genotyping using genomic DNA from the tail. All animal experiments were carried out with the approval of the Southern Medical University Animal Care and Use Committee in accordance with the guidelines for ethical treatment of animals [20].

### 4.2. Rapamycin Application

Two independent experiments were performed. Each experiment involved 10 mice that were able to eat independently by 3 weeks after birth; 10 mice were daily gavaged with rapamycin at 2 mg/kg body weight for 4 weeks; and the other 10 mice were treated with vehicle [20].

### 4.3. Radiographic Imaging and Skeletal Preparation

For radiographic analysis, 1-month-old mice were anesthetized with lidocaine and imaged at 10–50 kV for 30 s using LX-60 Faxitron Specimen Radiography System (Faxitron X-Ray Corporation, Lincolnshire, IL, USA). For skeletal preparation, whole-mount skeletal preparations of 8-week-old WT and KO mice were prepared by removing the skin and internal organs of the mice before immersion in 95% ethanol overnight. Specimens were stained with 0.015% Alcian Blue 8GX (Sigma Aldrich, Shanghai, China) in 80% ethanol/20% acetic acid, and 0.005% Alizarin Red (Sigma Aldrich, Shanghai, China) in 1% KOH after digestion with 2% KOH overnight. Specimens were cleared in a 1% KOH/20% glycerol solution and stored in a 1 : 1 mix of glycerol and 95% ethanol [20].

### 4.4. Micro-CT Skeletal Analysis

For micro-CT, individual vertebrae were fixed in 10% neutral buffered formalin and transferred to 70% ethanol. Imaging of the thoracic (T11) to lumbar (L5) vertebrae was performed using a micro-CT imaging system (ZKKS-MCT-Sharp-III scanner; Caskaishen, China). A small field was selected for scanning and was corrected for the CT value, with a 70 kV scanning voltage, 30 W, 429 *μ*A current, and 5 *μ*m scan thickness. A region of interest for quantitative analysis of trabecular bone was defined, extending from the proximal to the distal end of the vertebrae. For each sample, bone volume fraction (BV/TV), trabecular number (Tb.N), trabecular thickness (Tb.Th), trabecular separation (Tb.Sp), and height were measured. The 3D-MED 3.0 was used for three-dimensional knee reconstruction and image capture [20].

### 4.5. Histological Analysis

For histology, formalin-fixed samples were decalcified in 20% EDTA (pH 7.2) for 3 weeks, dehydrated through a graded series of ethanol, cleared, and embedded in paraffin. Five-micrometer sagittal sections were cut and stained with H&E or safranin O-toluidine blue [20].

### 4.6. Western-Blotting

Immunoblotting was performed as described in our previous studies [17]. To lyse cartilage, tissue was frozen and ground into a powder using liquid nitrogen in a mortar and the protein was collected. Protein was separated on 10% sodium dodecyl sulphate-polyacrylamide gels and transferred to nitrocellulose filter (NC) membranes (Millipore, Bedford, MA, USA). The membranes were incubated for 1 h with 4% dry skimmed milk in PBS buffer to block nonspecific binding. The membranes were then incubated with rabbit antibodies against Ps6 (1 : 1000; Cell Signaling Tech, CA, USA) and beta-actin (1 : 1000; Cell Signaling Tech, CA, USA). The membranes were then incubated with goat anti-rabbit or anti-mouse peroxidase conjugated secondary antibody (1 : 1000; Santa Cruz Biotechnology) for 1 h. The blots were visualized by enhanced chemiluminescence (ECL; Santa Cruz Biotechnology) using Kodak X-OMAT LS film (Eastman Kodak, Rochester, NY). All the Western-blotting tests were repeated at least 3 times [20].

### 4.7. Statistical Analysis

All quantitative data are expressed as mean ± SD. Statistical analysis was performed using independent samples *t*-tests and One-Way ANOVA. *p* < 0.05 was considered statistically significant. The statistical analyses were performed with SPSS 13.0 (IBM, Chicago, IL, USA) [20].

## 5. Conclusion

We established chondrocyte-specific TSC-1 KO mice to overactivate the energy metabolic component, mTORC1, and measured the spinal development by general, imaging, and histological assessments. The results showed that, in addition to skeletal dysplasia, the KO mice displayed severe congenital spinal kyphosis and significant intervertebral disc changes. This study suggested that, in the process of endochondral ossification, excessive activation of mTORC1 signaling in chondrocytes induces obvious spinal deformity, and the chondrocytes are the cell type responsible for congenital spinal deformity.
